# Bayesian cluster analysis

**DOI:** 10.1098/rsta.2022.0149

**Published:** 2023-05-15

**Authors:** S. Wade

**Affiliations:** School of Mathematics and Maxwell Institute for Mathematical Sciences, University of Edinburgh, James Clerk Maxwell Building, Edinburgh, UK

**Keywords:** Bayesian analysis, clustering, ensembles, mixture models, model misspecification

## Abstract

Bayesian cluster analysis offers substantial benefits over algorithmic approaches by providing not only point estimates but also uncertainty in the clustering structure and patterns within each cluster. An overview of Bayesian cluster analysis is provided, including both model-based and loss-based approaches, along with a discussion on the importance of the kernel or loss selected and prior specification. Advantages are demonstrated in an application to cluster cells and discover latent cell types in single-cell RNA sequencing data to study embryonic cellular development. Lastly, we focus on the ongoing debate between finite and infinite mixtures in a model-based approach and robustness to model misspecification. While much of the debate and asymptotic theory focuses on the marginal posterior of the number of clusters, we empirically show that quite a different behaviour is obtained when estimating the full clustering structure.

This article is part of the theme issue ‘Bayesian inference: challenges, perspectives, and prospects’.

## 1. Introduction

Clustering is one of the canonical forms of unsupervised learning, which aims to divide data points into *similar* groups, and has been used in various applications. Examples include astronomy to discover types of stars by clustering astrophysical measurements [1,2]; geosciences to detect minefields or seismic faults from spatial data [3]; natural language processing where topic models employ clustering to infer latent topics across documents for information retrieval [4]; biomedicine to discover groups of individuals or genes with similar patterns in gene expression or omics data [5,6] and many more. In many applications, the allocations and patterns within each cluster are of direct interest, while in other settings, clustering may be used in data preprocessing or feature engineering, or it may be used, not to recover homogeneous sub-populations, but, rather, as a building block in kernel methods for flexible density estimation or regression [7].

Algorithmic approaches, such as hierarchical, partition-based or density-based clustering, are commonly used in clustering. Hierarchical clustering builds a tree of clustering solutions, either through an agglomerative (bottom-up) and divisive (top-down) strategy [8,9], and results crucially depend on the choice of dissimilarity and linkage. Partition-based algorithms, including k-means [10] and k-mediods [9], aim to divide data into subsets by minimizing a specified loss function. In contrast to hierarchical algorithms, they provide a single clustering solution which is revisited and iteratively optimized. While the k-means algorithm is by far the most popular tool for clustering [11], there are several drawbacks (e.g. only covers numerical variables, sensitive to local optimum, requires the number of clusters k to be pre-specified). Lastly, density-based algorithms, such as DBSCAN [12,13], are based on the general idea of defining a cluster as a connected dense component. They are capable of discovering clusters of arbitrary shapes but lack interpretability. Although such algorithmic approaches are widely used, they are largely heuristic and not based on formal models, prohibiting the use of statistical tools, for example, in determining the number of clusters, and they lack measures of uncertainty in the clustering solution.

An alternative approach is model-based clustering, which uses mixture models, where each (non-empty) mixture component corresponds to a cluster [14–16]. Problems of determining the number of clusters and the component probability distribution can be dealt with through statistical model selection, for example, through various information criteria. The expectation–maximization (EM) algorithm is typically used for maximum likelihood estimation (MLE) of the mixture model parameters, consisting of the prior group probabilities and the local parameters of each component probability distribution. Given the MLEs of the parameters, the posterior probability that a data point belongs to a group can be computed through the Bayes rule. The cluster assignment of the data point corresponds to the component with maximal posterior probability, with the corresponding posterior probability reported as a measure of uncertainty. Importantly, however, this measure of uncertainty ignores uncertainty in the parameter estimates. As opposed to MLE, Bayesian mixture models incorporate prior information on the parameters and allow one to assess uncertainty in the clustering structure unconditional on the parameter estimates.

In this article, we provide a review of Bayesian approaches to clustering, including both model-based and loss-based methods (§2), along with an illustrative application to highlight the advantages of the Bayesian approach in §3. In addition, §4 brings together recent research on estimating the number of clusters and robustness to model misspecification in the model-based approach. This literature highlights the fundamental trade-off of mixture models between density estimation and clustering. As a simple solution, we discuss how one can separate the task of clustering by framing it in a decision-theoretic context. Importantly, this allows the mixture model to retain optimal statistical properties for density estimation, while also providing more robust clustering estimates.

## 2. Bayesian cluster analysis

In the context of clustering, the observed data consists of measurements $y=(y_{1},\ldots ,y_{n})$ drawn from a heterogeneous population consisting of an unknown number of homogeneous sub-populations. The observed $y_{i}\in Y$ may be continuous, discrete, mixed or more complex in nature (e.g. functional data). Each data point is associated with a discrete latent variable $z_{i}$ (also called the allocation variable) indicating the group membership of the data point, i.e. $z_{i}=j$ if $y_{i}$ belongs to the jth group, and $z_{i}=z_{i^{\prime }}$ if $y_{i}$ and $y_{i^{\prime }}$ belong to the same sub-population. We are interested in obtaining estimates of clustering structure characterized by the latent $z=(z_{1},\ldots ,z_{n})$ as well as describing the patterns within each cluster and understanding uncertainty in clustering structure. To achieve this, the Bayesian approach constructs a posterior distribution over clusterings, π(z|y)∝p(y∣z)π(z), where π(z) represents the prior over the space of clusterings and p(y∣z) can be defined through a model-based (§2a) or a loss-based (§2b) approach.

It is worth emphasizing that clustering is often referred to as an ill-posed problem, as it aims to discover unknown patterns or structures in the data. The notion of a cluster depends on the application at hand and can often be challenging to characterize formally. A unique clustering solution often does not exist [17]. Thus, one must carefully consider the model or loss employed and, importantly, also characterize uncertainty in the clustering solution. To achieve the latter, Bayesian cluster analysis provides a formal framework through both the posterior distribution over the entire space of clusterings and by creating an ensemble of clustering solutions sampled from the posterior. Moreover, this also helps to mitigate sensitivity to local optima which adversely impact all clustering algorithms due to the sheer size of the space.

As a note, in this article, we focus on clustering based on a single dataset. However, the massive growth in data acquisition and technologies has led to a number of interesting extensions. This includes combining multiple data sources through data integration [18–20], hierarchical Bayesian frameworks for partially exchangeable or nested data [21–28], hidden Markov models and other extensions for temporal data [29,30], accounting for spatially indexed data [31–33], incorporating general covariate information [34–37] and more.

### (a) Model-based approach

The most popular approach to Bayesian clustering employs a model-based framework through mixture models [38,39]. In this case, the data are assumed to be conditionally i.i.d. from a convex combination of parametric components:

2.1 $$y_{i}|w,\theta ,\psi \overset{\text{iid}}{∼}\sum_{j=1}^{J}w_{j}f(\cdot |\theta_{j},\psi )=\int f(\cdot |\theta ,\psi )\;dH(\theta ),$$

where f(y∣θ,ψ) is a fixed parametric density, often referred to as the kernel, with component-specific parameters contained in $\theta =(\theta_{1},\ldots ,\theta_{J})$ and global parameters ψ, and the mixture weights $w=(w_{1},\ldots ,w_{J})$ are non-negative and sum to one. In the equivalent integral representation on the right-hand side of (2.1), $H=\sum_{j=1}^{J}w_{j}\delta_{\theta_{j}}$ represents the mixing measure. Yet another equivalent representation, useful for clustering, makes use of allocation variables z:

$$y_{i}|z_{i}=j,\theta_{j},\psi \overset{\text{ind}}{∼}f(\cdot |\theta_{j},\psi ),\;z_{i}\overset{\text{iid}}{∼}\text{Cat}(w_{1},\ldots ,w_{J}),$$

where Cat(⋅) represents the categorical distribution with parameter w. In the Bayesian setting, the model is completed with a prior on the unknown parameters w, θ and ψ (or equivalently on the unknown mixing measure H and ψ).

In order to obtain clusters of practical relevance, the kernel f(⋅|θ,ψ) should be carefully selected to reflect the shape and properties of a cluster for the application at hand. A standard choice is the multivariate Gaussian distribution, $f(\cdot |\theta ,\psi )=N(\cdot |\mu_{j},\Sigma_{j})$. In fact, the widely used k-means algorithm can be seen as a limiting case of the EM algorithm for Gaussian mixture models, where the kernel is $N(\cdot |\mu_{j},\sigma^{2}I)$ [40,41]. This highlights that k-means imposes restrictive cluster shapes, specifically, all clusters have the same spherical shape of equal size in all dimensions, with only the centres $\mu_{j}$ allowed to differ across clusters. More generally, Gaussian mixture models relax this assumption by allowing different ellipsoidal shapes and sizes across clusters. The cluster-specific covariance matrices can be parametrized as $\Sigma_{j}=\lambda_{j}D_{j}A_{j}D_{j}^{T}$, where $\lambda_{j},D_{j}$ and $A_{j}$ control the volume, orientation and shape, respectively, of the ellipsoid and each parameter can be cluster-specific or global for general geometric cross-cluster constraints [14,42]. Other types of constraints on the covariance matrices can also be considered, such as mixtures of factor analysers [43,44] and mixtures of Gaussian graphical models within a casual framework [45,46].

However, depending on the data characteristics and aim, different kernels are more appropriate. For continuous data, skewed shapes and/or robustness to outliers can be accounted for through multivariate skew-normal or t-distributions [47,48], shifted asymmetric Laplace distributions [49] and normal-inverse Gaussian distributions [50]. For directional data on the unit sphere, examples include mixtures of Kent distributions [51], von-Mises–Fisher distributions [52] or Gaussian distributions in distinct tangent spaces [53]. For discrete data, mixtures of Bernoulli or multinomial distributions, known as latent class models, are appropriate for categorical data [4,54]; latent variable approaches using a logistic or probit transformation are employed for ordinal data [55,56]; and mixtures of Plackett–Luce models are used for rankings [57]. For count data, examples include mixtures of Poisson distributions [58,59], negative-binomial distributions [60] and rounded continuous kernels [61,62], as well as zero-inflated Poisson or negative-binomial distributions for sparse counts [63]. Mixed data of different types can be modelled by assuming either conditional independence, combining appropriate kernels through a product operation, or through a latent variable approach [64,65]. Moreover, the kernels may be themselves mixtures for increased flexibility [66,67].

In high-dimensional settings, challenges arise from both a computational [68] and a theoretical [69] perspective. In particular, Chandra *et al.* [69] show that one needs to be extremely careful in specifying both the kernel and the prior on θ in high-dimensions; otherwise, the posterior can degenerate on extreme clustering structures. One solution to overcome this employs variable selection methods within the mixture model to identify relevant variables that are informative for clustering, either using spike-and-slab priors [70–73] or shrinkage priors [74,75]. An alternative approach is to incorporate dimension reduction methods within the mixture model. This includes cluster-specific dimension reduction methods to reduce the number of parameters, such as mixtures of factor analysers [76] or parsimonious Gaussian mixtures [77], as well as approaches that conduct clustering directly on the lower dimensional space [69]. While approaches mainly focus on incorporating linear dimension reduction, extensions based on nonlinear dimension reduction can also be considered [78].

### (b) Loss-based approach

Partition-based clustering algorithms aim to minimize a specific loss function and are widely adopted but lack any quantification of uncertainty in the clustering solution. To address this and bridge the gap between partition-based and model-based approaches, the recent work of Rigon *et al.* [79] employs a generalized Bayesian framework through the use of Gibbs posteriors [80]. Specifically, the generalized posterior is defined as

π(z|y)∝exp⁡(−λℓ(z,y))π(z),

where the loss function has the form $\ell (z,y)=\sum_{j=1}^{k}\sum_{i:z_{i}=j}D(y_{i},y_{j})$ with $y_{j}=\{y_{i}:i=z_{j}\}$ denoting the observations belonging to the jth cluster and $D(y_{i},y_{j})\geq 0$ quantifying the discrepancy of $y_{i}$ from the jth cluster. A simple example is the k-means loss which sets $D(y_{i},y_{j})=∥y_{i}-y_{j}{∥}^{2}$ (additional examples can be found in Rigon *et al.* [79]). Fixing the number of clusters k, the prior π(z) is chosen to be uniform over the set of partitions with k clusters. In this case, the maximum a posterior (MAP) estimator ${\hat{z}}_{\text{MAP}}={\text{argmax}}_{z}\pi (z|y)$ corresponds to minimizing the loss function, e.g. under the k-means loss, ${\hat{z}}_{\text{MAP}}$ is the k-means solution. This provides an important link to partition-based approaches but also a significant enhancement through the uncertainty quantification offered by the Bayesian framework. However, a drawback of Bayesian loss-based clustering is that assumptions defining the notion of a cluster are less explicit compared with the model-based approach.

In addition, we highlight other interesting work integrating algorithmic approaches within a Bayesian framework. This includes combining density-based methods with a Bayesian model-based approach [81]; Bayesian hierarchical clustering which builds a tree of hierarchical clustering solutions based on Bayesian nonparametric (BNP) mixture models [82–85] and Bayesian distance-based clustering based on pairwise distances between observations [86–89].

### (c) Priors

#### (i) Number of clusters

One of the most difficult and important questions in clustering regards the choice of the number of clusters. In the model-based approach, the distinction between the number of components J and the number of clusters k requires emphasis. In fact, there may be no observations allocated to some components in the mixture, with possibly very small or even zero weight $w_{j}$ for some j∈{1,…,J}. Thus, the number of components provides an upper bound, i.e. k≤J. In general, there are four approaches to infer the number of clusters:

(i) Model selection tools or information criteria can be used to compare the mixture model under different choices of J [90]; in this case, penalization for empty clusters is implicitly included, so that the number of components corresponds to the number of clusters.
(ii) Mixtures of finite mixtures (MFM) [91–93] extend the hierarchy of the model with a prior on the number of components.
(iii) Overfitted mixtures specify J as an upper bound on the number of clusters with a sparsity promoting prior on the weights, which implicitly defines a prior on the number of clusters [74,94–96].
(iv) BNP mixtures [97] assume J=∞ and can be viewed as a limiting case of overfitted mixtures, with the Dirichlet process (DP) mixture [98] being the most widely-used example.

In the first approach, uncertainty on the number of clusters is lost with model fits based on all other choices of J disregarded. Instead, the subsequent three approaches are more natural from a Bayesian perspective, as they provide a posterior on the number of clusters, reflecting uncertainty.

#### (ii) Weights

To specify the prior on the weights, the standard choice for finite mixtures is the symmetric Dirichlet distribution, $\left(w_{1},\ldots ,w_{J})∼\text{Dir}(\alpha ,\ldots ,\alpha \right)$, due to its conjugacy with respect to the categorical distribution for z. A small value of the parameter α promotes sparsity in the weights, and in the extreme case when α→0, all prior mass is placed on the vertices of the simplex, with all weight on a single component. In overfitted mixtures, this sparsity property is essential to effectively regularize and prune extra components [95]. The parameter α has an influential role, and van Havre *et al.* [96] develop a parallel tempering algorithm to explore different values of α. While asymmetric Dirichlet priors may also be considered, symmetry with respect to relabelling of the clusters no longer holds. More generally, other distributions beyond the Dirichlet may be considered, such as the Generalized Dirichlet distribution [99], multinomial Pitman–Yor (PY) process [100], BFRY priors [101], normalized jumps of a finite point process [102] or non-informative Jeffreys priors [103].

For infinite mixture models, the prior on the weights must be constructed carefully to ensure the infinite sequence of weights $\left(w_{1},w_{2},\ldots \right)$ sum to one. A popular construction is stick-breaking [104,105], with a discussion on choice of hyperparameters in Giordano *et al.* [106]. Alternatively, the weights can be marginalized with a prior defined directly on the partition of data points into clusters. Exchangeable partition probability functions (EPPFs) [107] are a natural class of priors that result from the basic assumptions of exchangeability and invariance with respect to cluster labels, leading to priors that only depend on z through the cluster sizes $n_{j}=\sum_{i=1}^{n}\text{1}(z_{i}=j)$. For example, the EPPF obtained from the DP [108] has the form

$$\pi (z)=\frac{\Gamma (\alpha )}{\Gamma (\alpha +n)}\alpha^{k}\prod_{j=1}^{k}\Gamma (n_{j}),$$

where α>0 is a hyperparameter reflecting prior belief in the number of clusters. While this form is simple, intuitive and computationally appealing, it places most prior mass on highly imbalanced clusters with only a single parameter α to control prior uncertainty. Thus, there has been increased interest in exploring priors in the wider class of EPPFs and beyond, such as the general class of Gibbs-type priors [109,110], which contain the EPPF of the DP, PY process [111] and MFM [91] as special cases. Other proposals [112–114] aim to mitigate the *rich-get-richer* property of the DP to prefer highly imbalanced clusters; however, exchangeability often no longer holds. Subjective priors can also be specified which further enrich the parameter space by centring around prior information on the clustering structure [115,116]. In general, a BNP prior can be placed directly on the mixing measure H, which induces a prior on both the sequence of weights and the random partition. Indeed, the DP and PY are two widely used examples because they induce nice analytic priors for both the weights and partition. See Lijoi & Prünster [117] for an overview of priors beyond the DP.

#### (iii) Atoms

Often overlooked, the prior on the cluster-specific atoms $\theta =(\theta_{1},\ldots ,\theta_{J})$ also plays an important role, especially in high-dimensional settings. Typically, the atoms are assumed to be i.i.d. from a *base measure* $H_{0}$, i.e. $\theta_{j}\overset{\text{iid}}{∼}H_{0}$. A popular choice for $H_{0}$ is the conjugate prior to the kernel f(y|θ), which has the main advantage of computational convenience. The hyperparameters of the base measure can either be selected subjectively based on prior knowledge of the component-specific parameters, set empirically or inferred with additional hyperpriors; alternatively, data-dependent or non-informative priors can be used for $H_{0}$ [118]. For example, consider the Gaussian scale-location mixture with kernel $N(y|\mu_{j},\sigma_{j}^{2})$. The conjugate prior is the normal-inverse gamma:

$$\mu_{j}|\sigma_{j}^{2}∼N\left(\mu_{0},\frac{\sigma_{j}^{2}}{c}\right),\;\sigma_{j}^{2}∼\text{IG}\left(\frac{\nu }{2},\frac{\delta^{2}}{2}\right).$$

A data-dependent choice for $\mu_{0}$ is the empirical mean of the data. However, the scale parameter $\sigma_{j}^{2}$ represents the within cluster variance, and thus, the empirical variance provides an upper bound, where ν and δ should be carefully chosen to have most prior mass concentrated on values smaller than the empirical variance. As ν represents the degrees of freedom in the marginal t prior on $\mu_{j}$, Fraley & Raftery [119] suggest fixing ν to the smallest integer that gives finite variance, i.e. ν=d+2, and setting $\delta^{2}$ to be the empirical variance divided by ${\hat{k}}^{2}$, where $\hat{k}$ represents the prior guess on the number of clusters. The parameter c should be less than 1 to ensure higher between variance and can either be fixed, e.g. Fraley & Raftery [119] suggest a value of c=0.01, or assigned a hyperprior. Other examples of data-dependent priors can be found in Diebolt & Robert [120]; Richardson & Green [93]; and Wasserman [121]. We note that vague priors are not appropriate, as they will be highly influential on the posterior distribution, often favouring high within variance and one large cluster. In addition, while non-informative Jeffreys priors [122] for the atoms often lead to improper posteriors, non-informative priors can be combined with hierarchical hyperpriors to produce proper posteriors [123].

In order to favour components that are well separated, the independence assumption on the atoms can be relaxed through the use of repulsive priors [124–126], determinantal point processes [127] or non-local priors [128]. In particular, this form of prior regularization helps to improve interpretation and encourages more meaningful clustering structures, however, it also results in more complicated posterior computations. An alternative strategy is posterior regularization, which aims to find the variational solution with minimal Kullback–Leibler (KL) divergence to the posterior in a constrained space; this has been used to impose a max-margin constraint on DP mixtures [129,130] to ensure well-separated clusters.

## 3. Example: discovering cell subtypes

The rise in single-cell RNA sequencing (scRNA-seq) technology allows researchers to go beyond bulk RNA measurements and understand gene expression patterns at the single-cell level. Cells are heterogeneous in nature, and with existing technology able to record measurements on thousands of cells across thousands of genes, clustering has become an important tool to characterize latent cell types with similar expression patterns and summarize the data [131,132].

To highlight the advantages of Bayesian cluster analysis, we consider an experimental scRNA-seq dataset [133]^1^ collected to shed light on the development and fates of embryonic cells and the importance of the transcription factor PAX6 in the process. More generally, a single cell develops into an estimated 30 trillion cells in humans, and there is great interest in using single-cell technology and data to understand this process. In particular, PAX6 plays an important role in early development, and to empirically study this phenomenon the experimental data was collected at day E13.5 from mouse embryos under control (HET) and mutant (HOM) conditions in which PAX6 has been deleted. In the following, we present highlights of the analysis and results found in [60], which employs Bayesian model-based clustering to identify cell types, investigate how cell-type proportions change when PAX6 is knocked out, and explore if there are unique patterns when PAX6 is not present.

**Figure 1.**
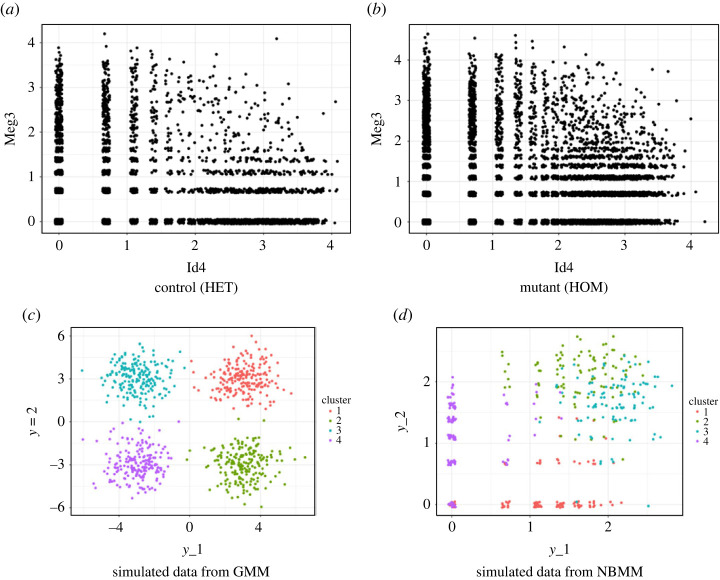
In order to highlight limitations of the standard workflow for scRNA-seq data, which firsts log-transforms data and then applies tools, such as k-means for clustering, we plot in (*a*,*b*) the log-transformed counts across all cells for two genes, Id4 and Meg3, and in (*c*) data simulated from a Gaussian mixture model (GMM); incompatibility and different characteristics are clearly observed between the real data (*a*,*b*) and simulated data (*c*). Instead, (*d*) plots log-transformed data generated from a negative-binomial mixture model (NBMM), which more closely resembles the real data. (Online version in colour.)

Most approaches for clustering scRNA-seq data separate the workflow into the steps of global normalization, dimension reduction and clustering. In particular, the data are often simply log-transformed, after adding an offset to avoid taking the logarithm of zero, and normalized in order to apply standard statistical tools. Figure 1 displays the log-transformed counts for two genes, Id4 and Meg3, and compares to data simulated from a spherical Gaussian mixture model. While it is standard to apply heuristic algorithms, such as k-means, to the log-transformed data, the incompatibility between the transformed data and the spherical clusters implied by k-means is clearly evident in figure 1. Instead, as discussed in §2a, the kernel in a model-based approach (or loss in a loss-based approach) should be more carefully considered to reflect the notion of a cluster. Moreover, the data are typically further transformed through dimension reduction methods, making the interpretation and specification of the notion of a cluster even more challenging. Indeed, as shown in Prabhakaran *et al.* [134] and Vallejos *et al.* [135], separating the workflow into normalization, dimension reduction and clustering can adversely affect the analysis, resulting in improper clustering and characterization of cell types.

Instead, the methodology and analysis of Liu *et al.* [60] follows more recent proposals which integrate normalization and clustering in a combined model-based framework [63,134,136,137]. Not only does this allow simultaneous recovery of clusters, inference of cell types and normalization, but it also provides measures of uncertainty that are propagated through the model hierarchy and coherent Bayesian updating. Importantly, the model-based approach allows for a more explicit definition of a cluster through careful specification of the kernel. Specifically, a negative-binomial kernel is employed to directly account for the count nature and overdispersion present in scRNA-seq data; that is the kernel is assumed to factorize across genes and for each gene is $\text{NB}(y|\beta \mu_{j},\phi_{j})$, where $\mu_{j}$ and $\phi_{j}$ represent the cluster-specific mean expression and dispersion and β is the cell-specific capture efficiency, representing the fraction of transcripts recovered. For more robust estimates in the case of sparse data or small clusters, a hierarchical prior for the atoms $\left(\mu_{j},\phi_{j}\right)$ is used that accounts for the mean-variance relationship [138]. Borrowing of strength and shared clustering across the mutant and control conditions is permitted through a hierarchical Bayesian framework, namely the hierarchical DP [28]. For full details, see [60].

**Figure 2.**
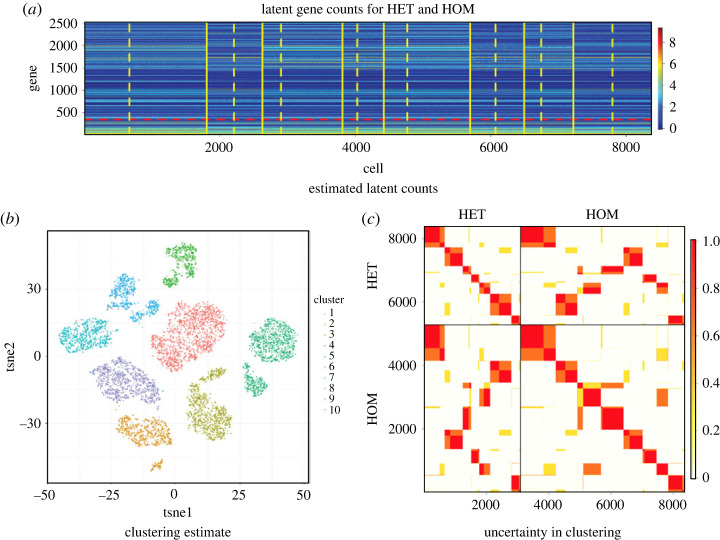
Highlights of the analysis of Liu *et al.* [60]. (*a*) Heat map of the posterior estimated latent RNA counts (corrected by the posterior capture efficiencies) for each cell (x-axis) and gene (y-axis). Cells from different clusters are separated by solid yellow lines, and within each cluster, the dashed yellow line separates HOM and HET. Genes above the red horizontal line are identified as differentially expressed across the clusters. (*b*) Visualization of the clustering estimate in the two-dimensional space obtained through t-distributed stochastic neighbour embedding (t-SNE [139]) of the high-dimensional data. (*c*) Uncertainty in clustering characterized by the posterior similarity matrix. (Online version in colour.)

The Bayesian approach permits us to produce a range of graphical tools and tables to visualize and summarize not only point estimates but also uncertainty in the clustering structure and all parameters. The posterior estimated latent counts (corrected by the posterior capture efficiencies) are shown in figure 2*a*. Solid yellow lines separate the cells by cluster, and within each cluster, the dashed yellow line separates cells from the HOM and HET conditions. When focusing on the latent counts and genes identified as differently expressed, the clusters are visually well separated (figure 2*b*). In addition to the clustering estimate, we can also visualize uncertainty in the clustering structure, for example through the *posterior similarity matrix*, whose elements represent the posterior probability that two cells are clustered together. While the blocks of red highlight evident clusters of cells with posterior probability close to one, there are also some cells with more uncertainty in their allocation. Alternative tools to visualize and describe uncertainty through credible balls are provided in Wade & Ghahramani [140]. In summary, the model estimates a total of eight clusters, which are all shared in the control and mutant conditions (with some uncertainty on further splitting some clusters). Certain clusters are under or over represented in the mutant condition when PAX6 is knocked out, and further discussion on the results can be found in [60].

## 4. Estimating the number of clusters and model misspecification

In this last section, we bring together recent research on estimating number of clusters, with a focus on the debate between finite mixtures (MFM, overfitted) versus infinite mixtures (BNP), and robustness to model misspecification. Infinite BNP mixtures assume that the number of clusters depends on the sample size and grows unboundedly as more data are collected. With advancements in computing and general inference schemes such as Markov chain Monte Carlo (MCMC), BNP mixtures can be easily implemented. Moreover, well-established theory validates the use of BNP mixtures for asymptotically optimal density estimation [141–144]. Together these properties and developments have led to the huge growth and adoption of BNP mixtures, especially DP mixtures, for a variety of applications in statistics and machine learning in the twenty-first century.

However, this enthusiasm was dampened by the negative results of Miller & Harrison [145,146] that provided a simple example in which the posterior on the number of non-empty components in DP mixtures is inconsistent when true number is finite. In fact, the posterior is demonstrated to be *severely* inconsistent, as the posterior probability that the number of non-empty components equals the truth asymptotically tends to zero. This is in contrast to overfitted mixtures, which asymptotically prune extra components [95,118], and MFM which yield consistent estimates for the number of components [147,148]. While overfitted mixtures can be viewed as truncated approximations to DP mixtures, this seemingly contradictory result can be explained by noting that BNP mixtures are misspecified when the true number of components is finite, and in this case, the true density lies at the boundary of the prior support [147]. Indeed, it is well-known that DP mixtures can introduce many small extra clusters. To overcome this, Guha *et al.* [147] develop a post-processing procedure to consistently estimate the true number of components by suitably truncating components with small weights and merging similar components. Instead, consistency can also be achieved by adapting the concentration parameter α of the DP to be sample-size dependent or via a suitable hyperprior, which is in fact standard in practice [149,150]. Furthermore, Frühwirth-Schnatter & Malsiner-Walli [151] illustrate that the choice of the hyperprior on the weights is far more influential on the number of clusters than whether an overfitted or DP mixture is considered.

In practice, we can expect that mixture models are misspecified in some way; either in the kernel or mixing measure, or both. Optimal asymptotic results of Bayesian mixtures for density estimation still hold (in the sense of convergence to the KL projection of the true density into the prior’s support) [152,153]. However, Guha *et al.* [147] show that mild misspecification leads to very slow contraction rates of the mixing measure (with respect to its KL projection) and that the choice of the kernel is especially important; moreover, BNP mixtures are better suited to adapt to complex forms of the density in the misspecified setting. Cai *et al.* [154] also show that for MFM, even slight model misspecification leads to inconsistency for the number of clusters. As mixture models are inherently built for density estimation, it is intuitively reasonable that an overestimation of the number of clusters occurs in the misspecified case, since more components are required to accurately recover the density. This highlights the fundamental trade-off between clustering and density estimation for mixtures.

To improve model-based clustering in the misspecified setting, robust clustering methods have been developed. Examples include coarsened posteriors for mixture models [155] as well as modal-based clustering [156]. While such approaches may result in more robust clustering solutions, optimal statistical properties for density estimation may be lost. In general, both density estimation and clustering may be of interest. Thus, we consider Bayesian model-based clustering via mixtures, to retain optimal properties for density estimation, and focus on the comparison of different estimates for the number of clusters and clustering solution. In fact, we find that the number of clusters can change drastically depending on the estimator used.

The literature discussed above estimates the number of clusters via the marginal posterior on the number of non-empty components. Alternatively, the full clustering solution can be estimated, without conditioning on the number of clusters, thus also implicitly providing an estimate of this number. In fact, Rajkowski [157] demonstrates that the MAP clustering has desirable asymptotic properties in the simple example of Miller & Harrison [145], in stark contrast to the severe inconsistency of the marginal posterior on the number of clusters. To estimate the clustering solution, various *ad hoc* methods have been proposed [158–161]. Instead, we focus on a decision-theoretic approach, obtaining the optimal clustering by minimizing the posterior expectation of a specified loss function measuring the discrepancy between the true and estimating clustering. The MAP clustering is obtained under the 0–1 loss, and various search algorithms have been developed to locate the MAP solution [82,162–164]. Alternative loss functions were considered in Fritsch & Ickstadt [165]; Lau & Green [166]; Quintana & Iglesias [167] and Wade & Ghahramani [140]. Two widely used loss functions, which are considered below, are Binder’s loss^2^ [168] and the variation of information (VI) [169]. General algorithms to optimize the posterior expected loss can be found in [140,166], with more recent schemes in Dahl & Müller [170], Dahl *et al.* [171] and Rastelli & Friel [172] that are particularly suited to large sample sizes and parallel computations.

To illustrate the differences between the estimators, we consider two simple examples: data generated from (i) a standard normal distribution and (ii) a uniform distribution on the unit circle, and in both cases, a DP location-scale mixture of Gaussians is employed for model-based clustering. The famous example of Miller & Harrison [145] corresponds to (i) and the marginal posterior on the number of clusters was demonstrated to be severely inconsistent. Rajkowski [157] considered the second example and proved that when the within cluster variance is set too small (in a DP location mixture of Gaussians with fixed within cluster variance), the MAP clustering is not unique and partitions the ball into several, seemingly arbitrary convex sets. In all experiments, the posterior is approximated via MCMC with 10 000 iterations. For each example, 50 replicated datasets are generated and different sample sizes of n=100,200 and 500 are considered. Sensitivity to the choice of DP concentration parameter α is explored, with α=0.5,1 and 2 and a sample-size dependent choice of α=1/log⁡(n). The same search algorithm is performed for the MAP, Binder and VI estimates; specifically, first, we select the clustering which minimizes the posterior expected loss among both the MCMC draws and the set of clusterings obtained through a hierarchical clustering algorithm (with dissimilarity equal to one minus the posterior similarity), and then, we perform a greedy search [140] starting from this clustering for any possible further improvements.

**Figure 3.**
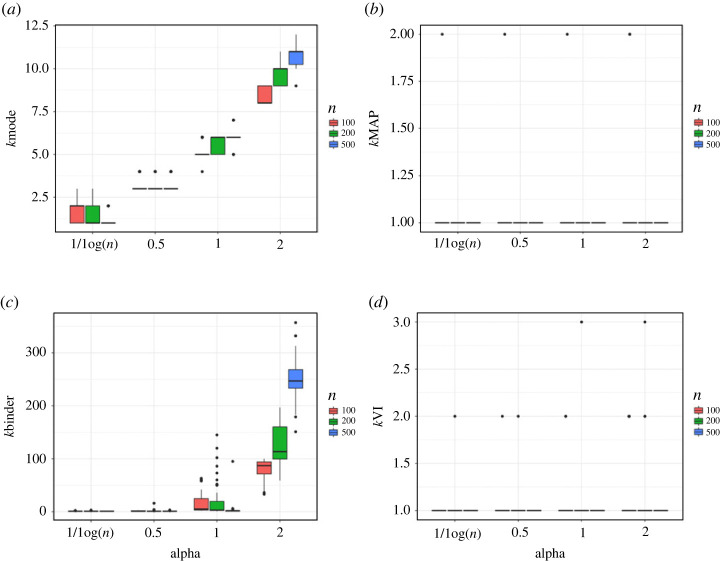
Comparison of different estimators for the number of clusters in the example of Miller & Harrison [145], where the true clustering contains only a single cluster. The DP mixture of Gaussians is considered for model-based clustering with different choices of the concentration parameter α. The box plots display variability in the estimates across the 50 replicated datasets, with colour corresponding to a sample size of n=100,200 or 500. (*a*) Marginal mode of k. (*b*) MAP clustering k. (*c*) Binder clustering k. (*d*) VI clustering k. (Online version in colour.)

Figure 3 compares the different estimators for the example of Miller & Harrison [145]. Focusing on the mode of the marginal posterior on k (figure 3*a*), we empirically observe the inconsistency shown by Miller & Harrison [145]. Results are also sensitive to the choice of α, and the sample-size dependent choice of α=1/log⁡(n) empirically helps to mitigate this behaviour (as expected based on Ascolani *et al.* [149] and Ohn & Lin [150]). Instead, the MAP clustering (figure 3*b*) contains only a single cluster (as proved by Rajkowski [157]) and is robust to the choice of α. Depending on α, the DP mixture tends to create small extra clusters at each iteration. As discussed in Wade & Ghahramani [140], Binder’s loss has a preference to split off small clusters over merging, and thus, when α is too large, the Binder clustering (figure 3*c*) extremely overestimates the number of clusters. The VI is a more symmetric metric in this regard, and therefore, the VI clustering (figure 3*d*) only contains a single cluster, in almost all replicates, that is also robust to the choice of α.

**Figure 4.**
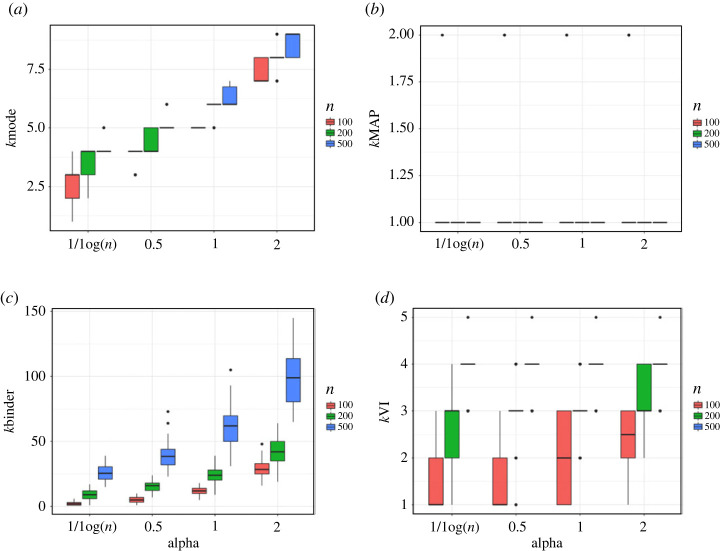
Comparison of different estimators for the number of clusters in the misspecified example of Rajkowski [157], where the true clustering contains only a single cluster under the uniform kernel. The DP mixture of Gaussians is considered for model-based clustering with different choices of the concentration parameter α. The box plots display variability in the estimates across the 50 replicated datasets, with colour corresponding to a sample size of n=100,200 or 500. (*a*) Marginal mode of k. (*b*) MAP clustering. (*c*) Binder clustering. (*d*) VI clustering. (Online version in colour.)

Results for the example of Rajkowski [157] are shown in figure 4. This is a misspecified example; while the true clustering under the uniform kernel contains only a single cluster, the DP mixture of Gaussians explores various arbitrary partitions of the data to approximate the uniform distribution. While Rajkowski [157] found that the MAP clustering also partitions the unit circle into several arbitrary sets when the within cluster variance is fixed and set too small, we, however, empirically observe a different behaviour when incorporating uncertainty on the within cluster variance. In fact, the MAP clustering (figure 4*b*) contains only a single cluster in almost all replicates and is robust to the choice of α. Again, the marginal posterior on k (figure 4*a*) and the Binder clustering (figure 4*c*) are quite sensitive to the value of α, with the Binder clustering extremely overestimating the number of clusters for larger n and α. The VI clustering (figure 4*d*) contains only a single clustering in some replications, and in others contains two to four clusters, particularly for larger sample sizes. The former can be explained by the fact that the VI solution is obtained by minimizing a function of the posterior similarity matrix, and as each posterior sample corresponds to an arbitrary partition of the data points into convex sets, each pair of data points may have a relatively high probability of being clustered together.

These examples highlight that the choice of estimator can greatly affect the number of clusters and clustering solution. In fact, while most asymptotic theory focuses on the behaviour of the marginal posterior on the number of clusters, quite a different behaviour is observed when estimating the full clustering solution. As practitioners are interested in the full clustering solution, this is an important aspect to consider. There are a number of interesting directions to expand this study, including further investigating the performance of the different estimators in the misspecified setting, as well as the case when the clusters are not well separated (Rajkowski [157] finds that MAP tends to underestimate the number clusters in this setting), and sensitivity to hyperparameters. Other estimators can be studied, e.g. Dahl *et al.* [171] develops generalized forms of Binder’s loss and VI with unequal penalties, which provide more control over the estimated number of clusters but require specifying an additional parameter of the generalized loss. Moreover, this study can be expanded by empirically comparing different models (MFM, sparse mixtures and infinite mixtures beyond the DP), as well as quantifying uncertainty, e.g. through empirical coverage of credible balls around the estimators [140]. Finally, while we have focused on general, commonly used estimators, it must be emphasized that in applications more problem-specific estimators should also be considered. This is achieved by defining an application-specific loss function in the decision-theoretic framework; examples include clinical trials [173,174] and earthquake studies [175].

## 5. Conclusion

The article contains an overview of Bayesian cluster analysis, which offers substantial benefits over algorithmic approaches by providing not only point estimates but also uncertainty in all parameters. More specifically, through the posterior over the clustering structure, an ensemble of clustering solutions is obtained. This ensemble and associated uncertainty can be visualized and described through various graphical tools and quantities, such as the posterior similarity matrix, credible balls, cluster comparison criterion [169] and stability indices [176]. The benefits are showcased in an application to cluster cells and discover latent cell types in scRNA-seq data to improve understanding of embryonic cell development [60].

We have provided a review of two approaches to Bayesian cluster analysis: model-based and loss-based. In both, careful consideration of the kernel or loss is emphasized for clustering solutions of practical relevance. The Bayesian paradigm requires specification of priors over the unknown parameters. Most often this includes the number of clusters, and a review of relevant approaches is given.

Lastly, we have focused on the ongoing debate between finite and infinite mixtures in a model-based approach and robustness to model misspecification. While much of the debate and asymptotic theory has focused on the marginal posterior of the number of clusters, we have empirically shown that quite a different behaviour is obtained when estimating the full clustering solution. As the full clustering solution is required in applications, the results highlight that more emphasis should be placed on this aspect. All models are misspecified in some way, and while careful consideration of the kernel in mixture models helps, robustness to misspecification should be acknowledged. Mixture models are inherently built for density estimation; if robust clustering methods are employed, optimal density estimation is sacrificed. Instead, our simple experiments highlight that mixtures models can still be employed, to retain optimal density estimation, with robust clustering via separation of the clustering problem in a decision-theoretic framework and careful consideration of the loss and estimators used. The MAP and VI clustering solutions are general and provide robust estimates in the examples presented, but in applications, more problem-specific estimators should also be explored [173].

## Data Availability

The data are provided in electronic supplementary material [177].

## Footnotes

1 Collected and prepared by Dr Kai Boon Tan and the research group lead by Prof. David Price and Prof. John Mason at the Centre for Brain Discovery Science, University of Edinburgh.

2 Note that minimizing the posterior expected Binder’s loss is equivalent to maximizing the posterior expected Rand Index or Hamming distance.
